# The Clinical Effectiveness of Propolis on the Endodontic Treatment of Permanent Teeth: A Systematic Review of Randomized Clinical Trials and Updates

**DOI:** 10.7759/cureus.77430

**Published:** 2025-01-14

**Authors:** Amirah Y Aldosari, Amira M Aljared, Hanin S Alqurshy, Abdullah M Alfarran, Mohanad G Alnahdi, Sarah S Alharbi, Wed S Alharbi, Faisal T Alghamdi

**Affiliations:** 1 General Dentistry, Faculty of Dentistry, King Abdulaziz University, Jeddah, SAU; 2 General Dentistry, Faculty of Dentistry, Riyadh Elm University, Riyadh, SAU; 3 Oral Biology, Faculty of Dentistry, King Abdulaziz University, Jeddah, SAU

**Keywords:** bee wax, endodontics, intracanal medicament, propolis, pulp capping, randomized clinical trial, review

## Abstract

Endodontic treatment of permanent teeth (immature and mature) with propolis in healthy patients remains uncertain, with conflicting evidence. Therefore, this systematic review aims to evaluate the current literature on the safety and efficacy of propolis in endodontic procedures. An extensive literature search was performed using PubMed, Scopus, Web of Sciences, and Google Scholar. Only human randomized clinical trials (RCTs) that explored the clinical applications of propolis in endodontics were considered. Narrative synthesis was performed, and the risk of bias (RoB) for methodological quality assessment was performed with the Cochrane RoB-2 via the Robvis web-based application. Eight RCTs were selected, focusing on using propolis in vital pulps for direct pulp capping and non-vital pulps for root canal disinfection and filling materials. Propolis demonstrated promising effects, including controlling inflammation, promoting tissue healing, and disinfecting the root canal system. However, no significant differences were observed when comparing propolis to other materials used in pulp capping or intracanal medicaments. The RoB assessment revealed varied levels of risk, with two studies exhibiting a high risk, three having unclear risks, and three showing low risk. Moderate certainty of evidence was observed. Based on the current evidence, there is insufficient data to recommend propolis over other materials in the treatment of vital or non-vital pulps in permanent teeth. Propolis was not recommended as a definitive treatment due to the limited evidence and variability in the clinical outcomes across studies. However, future high-quality RCTs are essential for more definitive conclusions.

## Introduction and background

In endodontic therapy, managing vital and non-vital pulp conditions is essential, requiring approaches tailored to the pulp’s status [1,2]. Vital pulp therapy focuses on preserving viable tissue when the pulp remains healthy or minimally inflamed, utilizing techniques like pulp capping to shield the pulp from further injury [1]. Treatment for non-vital pulp, often caused by infection or trauma, typically removes necrotic pulp tissue through root canal therapy to prevent infection and restore function [2]. Likewise, root canal therapy for non-vital pulp includes cleaning, shaping, and sealing the canal space to create a sterile environment critical for the procedure’s success [3,4].

Proper instrumentation is fundamental to lowering bacterial loads and optimizing disinfection, thus enhancing the chances of successful treatment [5], as instrumentation techniques can significantly reduce the microbial presence and also create a clean environment that minimizes the risk of reinfections [6]. Irrigation plays an essential role in removing remaining debris bacterial toxins, and necrotic tissue within the canal [7]. Traditional irrigants like sodium hypochlorite (NaOCI) and chlorhexidine (CHX) are well-known for their bactericidal properties [8,9]. Alternative irrigants like propolis are being studied for their natural antimicrobial and biocompatible properties, especially in tackling resilient bacteria like *Enterococcus faecalis* (*E. faecalis*) [10,11].

Propolis is a bee product primarily composed of plant resins, essential oils, beeswax, and a variety of organic compounds [12]. It is particularly rich in compounds like flavonoids and phenolics, which have a known role as anti-inflammatory, antibacterial, and antioxidant properties [13]. These components make propolis valuable as a natural agent in various dental applications, particularly in endodontics, where it could serve as an alternative to synthetic chemicals [14].

Propolis mouthwash has significantly reduced the size and frequency of aphthous ulcers, shortened healing time, and decreased local discomfort [15]. It also helps to mitigate metabolic changes caused by periodontopathic bacteria, which can contribute to systemic diseases [16]. Propolis effectively reduces gingivitis, plaque, and dental caries, offering both therapeutic and preventive benefits [17,18]. It has been reported to enhance clinical outcomes in chronic periodontitis by improving probing pocket depth and clinical attachment levels [19]. Furthermore, propolis hydrogel combined with iontophoresis has been used as a natural alternative to fluoridated desensitizers [20]. Propolis also preserves periodontal ligament cell viability in avulsed teeth better than conventional solutions like Hanks' balanced salt solution (HBSS) and milk [21,22]. Similarly, when applied as an intracanal medicament during the endodontic treatment, propolis has been reported to effectively combat *E. faecalis*, especially when combined with calcium hydroxide (Ca(OH)2) and antibiotics like moxifloxacin and ciprofloxacin [23]. Propolis combined with mineral trioxide aggregate (MTA) has shown better outcomes regarding inflammation and significantly up-regulated dental pulp stem cell expression [24]. As a pulp capping agent, propolis significantly reduces pulpal inflammation, promoting the formation of high-quality tubular dentin without infection or necrosis [25]. Moreover, a zinc oxide-propolis mixture was used as a root canal filling material for primary teeth after 24 months, highlighting its versatility and reliability [26]. Together, these reports suggest a role for propolis in dental care.

Differences in research design, measuring methodologies, and study populations have contributed to varying results with propolis, making it difficult to make firm conclusions. Few reviews have attempted to reconcile these disparate findings [27-31]. Only one recent scoping review examined the therapeutic efficacy of propolis in endodontics [31]. Previous clinical studies have evaluated the use of propolis alone in endodontic therapy to reduce pulpal tissue inflammation and improve dentinal bridge development. Studies demonstrate clinical and radiographic success in endodontic treatment and reducing the risk of pulp inflammation through dentin bridge formation [32,33]. To our knowledge, no systematic review has been published comparing randomized clinical trials (RCTs) that evaluated the propolis clinical effectiveness in endodontics for mature and immature permanent teeth. This systematic review summarized the current literature on the safety and efficacy of propolis in endodontic procedures for both mature and immature permanent teeth. The review also explores the potential role of propolis in clinical practice within the field of endodontics.

## Review

Materials and method

This review was performed using Preferred Reporting Items for Systematic Reviews and Meta-Analyses (PRISMA) guidelines to prefer the most relevant RCTs [34]. The study protocol was registered (CRD42024606501) in the International Prospective Register of Systematic Reviews database.

*Literature* *Search* *Strategy*

The literature search strategy used the PICOS format: participants (P): healthy children, adult participants with mature/immature permanent teeth; intervention (I): use of propolis; comparator/control: other materials (irrigating solutions and filling materials) used in standard endodontic treatment; outcome (O): sufficient evidence for clinical or radiographic success of endodontic treatment by applying propolis. Study design (S): RCTs. The literature search was performed from inception to 10th November 2024 using the PubMed, Scopus, Web of Sciences, and Google Scholar search engines. The following MeSH and search terms were used: (("root canal therapy" "endodontic therapy" OR "endodontology" OR "endodontic" OR "intracanal dressing" OR "intracanal medicament" OR "pulp capping" OR "dental pulp" OR "vital tooth" OR " vital pulp" OR "necrotic pulp" OR "non-vital pulp" OR "pulp regeneration" OR "regenerative endodontics" OR "revascularization") AND ("propolis extract" OR "propolis" OR "honey-based material" OR "bee honey" OR "bee bread" OR "bee wax" OR "bee glue" OR "phenolic acids" OR "flavonoids")) as shown in Table 1.

**Table 1:** The search strategy employed in this study.

Database	Search strategy	Results
PubMed with no time restrictions–up to November 10, 2024	#1((((((((((((((((root canal therapy) OR (endodontic therapy)) OR (endodontology)) OR (endodontic)) OR (intracanal dressing)) OR (intracanal medicament)) OR (pulp capping)) OR (dental pulp)) OR (vital tooth)) OR (vital pulp)) OR (necrotic pulp)) OR (non-vital pulp)) OR (pulp regeneration)) OR (regenerative endodontics)) OR (revascularization)) AND #2 (((((((((propolis extract) OR (propolis)) OR (honey-based material)) OR (bee honey)) OR (bee bread)) OR (bee wax)) OR (bee glue)) OR (phenolic acids)) OR (flavonoids)) #3 #1 AND #2	#3=371
Scopus with no time restrictions–up to November 10, 2024	#S1"root canal therapy" OR "endodontic therapy" OR "endodontology" OR "endodontic" OR "intracanal dressing" OR "intracanal medicament" OR "pulp capping" OR "dental pulp" OR "vital tooth" OR " vital pulp" OR "necrotic pulp" OR "non-vital pulp" OR "pulp regeneration" OR "regenerative endodontics" OR "revascularization" AND #S2 "propolis extract" OR "propolis" OR "honey-based material" OR "bee honey" OR "bee bread" OR "bee wax" OR "bee glue" OR "phenolic acids" OR "flavonoids" #S3= #S1 AND #S2	#S3=278
Web of Science with no time restrictions–up to November 10, 2024	#TS1=("root canal therapy" OR "endodontic therapy" OR "endodontology" OR "endodontic" OR "intracanal dressing" OR "intracanal medicament" OR "pulp capping" OR "dental pulp" OR "vital tooth" OR " vital pulp" OR "necrotic pulp" OR "non-vital pulp" OR "pulp regeneration" OR "regenerative endodontics" OR "revascularization") AND #TS2=("propolis extract" OR "propolis" OR "honey-based material" OR "bee honey" OR "bee bread" OR "bee wax" OR "bee glue" OR "phenolic acids" OR "flavonoids") #TS3= #TS1 AND #TS2	#TS3=162
Google Scholar with no time restrictions–up to November 10, 2024	"root canal therapy" OR "endodontic therapy" OR "endodontology" OR "endodontic" OR "intracanal dressing" OR "intracanal medicament" OR "pulp capping" OR "dental pulp" OR "vital tooth" OR " vital pulp" OR "necrotic pulp" OR "non-vital pulp" OR "pulp regeneration" OR "regenerative endodontics" OR "revascularization" AND "propolis extract" OR "propolis" OR "honey-based material" OR "bee honey" OR "bee bread" OR "bee wax" OR "bee glue" OR "phenolic acids" OR "flavonoids"	237

*Eligibility* *Criteria*

Different inclusion criteria were set for the selection of studies. For instance, studies compared the use of propolis with other materials for endodontic irrigation or filling materials. Only RCTs performed on human subjects reported in English were evaluated.

Exclusion criteria included non-RCTs, observational studies, case reports, reviews, editorials, conference abstracts, editorials, and opinions regarding the use of propolis on primary teeth, ex vivo studies, animal studies, and in vitro studies.

*Studies* *Selection*

Six independent reviewers (AYA, AMA, HSA, AMA, MGA, and WSA) reviewed the search findings. Initially, studies were identified, and duplicates were removed. In the screening phase, titles and abstracts were screened, and relevant studies were included for full-text assessment based on the eligibility criteria. Disagreements were resolved through discussion. In case the issue was not resolved, two senior reviewers (SSA and FTA) provided the final decision. There was a Cohen's kappa inter-agreement score of 0.90 (labeled as “almost perfect”) for the study selection process.

*Data* *Extraction*

A systematic and structured approach was applied during the data extraction process using a predefined and standardized Microsoft Excel sheet (Microsoft Corporation, Redmond, Washington, USA) with the variables "title, abstract, methods, type of propolis used, type of teeth, type of clinical applications, follow-up period following the application of propolis, and main results."

*Data* *Items*

The extracted data from selected studies included the author's ID, country, sample size, tooth type (incisors, canines, premolars, and molars), age range of the participants, and details regarding the propolis formulation (paste, extract, mixed with saline/ethanol, or combined with other agents). Similar data was extracted regarding the comparison groups. Follow-up duration, assessment methods (including clinical and radiographic evaluations, visual analog scale (VAS) for pain intensity, or microbiological analysis), and key findings were documented.

*Methodological* *Quality* *and* *Risk* *of* *Bias* *Assessment*

The methodological quality of selected articles was determined using the Cochrane risk of bias (RoB)-2.0 tool with Robvis for visualization of the outcomes [35]. Three independent reviewers (AYA, SSA, and FTA) evaluated each study.

*Types* *of* *Outcome* *Measurements*

The primary outcomes of this review focus on studies that assess the success of propolis as a pulp capping material in permanent teeth with vital pulps. The success is measured using clinical parameters (e.g., symptom resolution and tissue healing) and radiographic evidence (e.g., dentin formation and absence of infection).

The secondary outcomes include studies evaluating the use of propolis as an intracanal medicament in non-vital permanent teeth. Success for these secondary outcomes is measured by factors such as the resolution of infection, healing of periapical tissues, and overall treatment success based on clinical and radiographic assessments.

*Data* *Synthesis*

A narrative approach was followed to summarize the extracted data in the form of tables. These tables include the study and participant characteristics of each included study. Due to heterogeneity between studies, no meta-analysis was performed. The Grading of Recommendations Assessment, Development, and Evaluation (GRADE) framework was used to rate the certainty of evidence [36].

*Grading of Recommendations Assessment, Development, and Evaluation (GRADE) Assessment* 

In our systematic review, the GRADE framework was employed to assess the certainty of the evidence [36]. The GRADE methodology evaluates evidence across several domains, including study limitations (risk of bias), consistency, directness, precision, and publication bias, which together inform the overall strength of the evidence. We used the GRADEpro Guideline Development Tool (GDT) software (GRADEpro 3.0 software, Windows-based, Evidence Prime Inc., Hamilton, Ontario, Canada) to facilitate this assessment. 

*Statistical* *Analysis* 

The studies included in our review exhibited significant heterogeneity, which was one of the reasons why a meta-analysis was not conducted. The heterogeneity arose from differences in the study populations, intervention protocols (such as varying dosages and forms of propolis), and outcome measures. Additionally, there were inconsistencies in the way some key parameters were reported, such as the origin and extraction methods of propolis, which could potentially influence the findings. This variability in study design and reporting contributed to the difficulty in performing a quantitative synthesis of the data. Thus, a narrative synthesis was employed to summarize the results across the studies. 

Results

*Study* *Selection*

**Figure 1:**
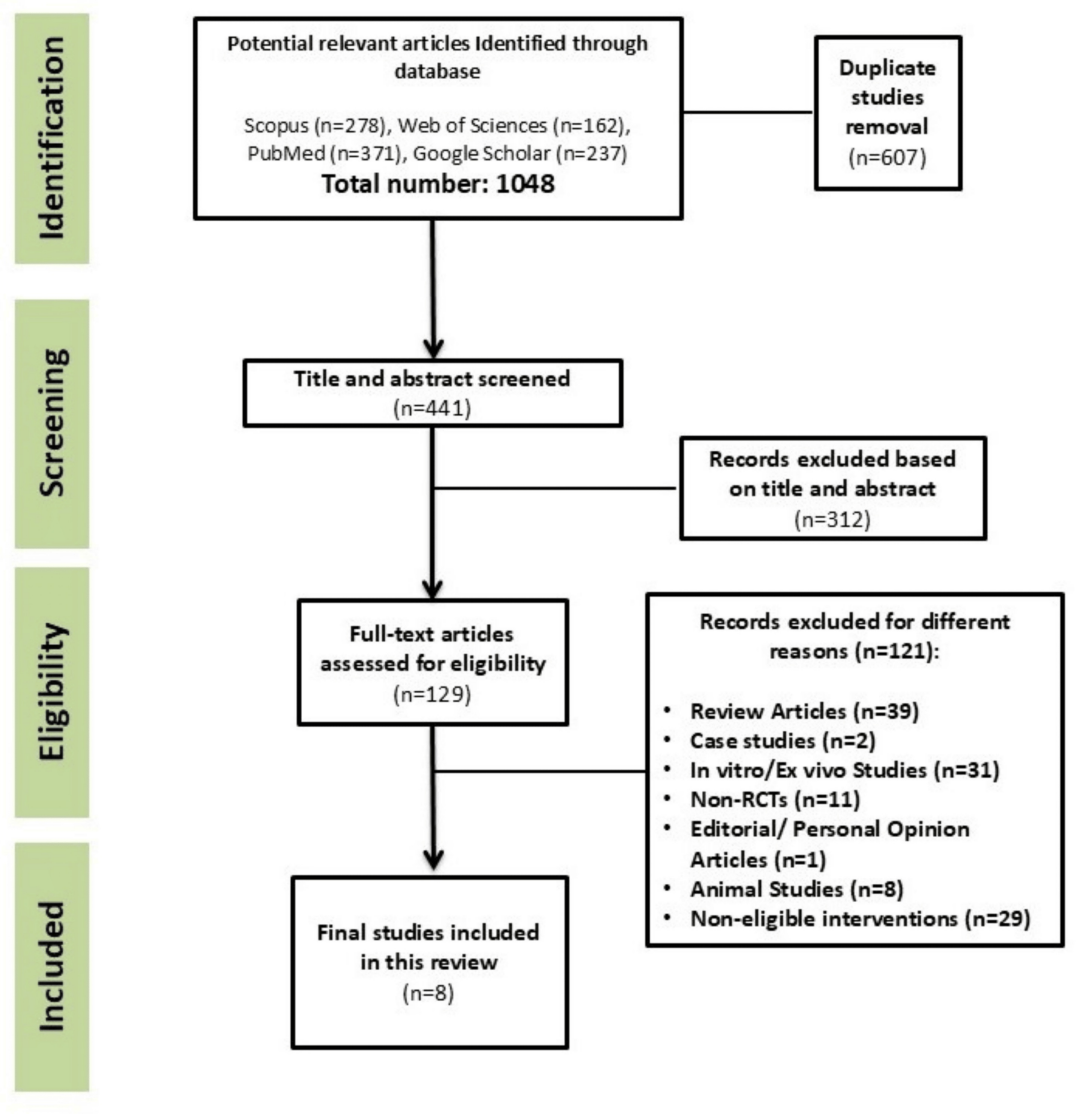
Flowchart for the selection of studies. RCTs: randomized clinical trials.

One thousand and forty-eight publications were identified using the search criteria (Figure 1). Six hundred and seven were duplicates and were excluded. Three hundred and twelve were excluded during the screening of the titles and abstracts (Figure 1). One hundred twenty-one publications were excluded due to not meeting eligibility criteria (Figure 1). Eight RCTs met the eligibility criteria for this systematic review.

Study Characteristics

All included studies were RCT studies (Tables 2, 3) [37-44]. Propolis was clinically applied as a direct pulp capping material in four RCTs [37-40] and an intracanal medicament in four RCTs [41-44]. Seven RCT studies were reported from Asian countries, including India [39-41,44], Pakistan [42,43], and Iran [38], and one RCT study was from an African country, Egypt [37]. The included RCTs involved 454 teeth of healthy patients ranging in age from 13 to 50 years. None of these eight RCTs reported patient gender.

**Table 2:** Details of clinically selected studies in which propolis is used as pulp capping material in permanent teeth. NR: not reported; MTA: mineral trioxide aggregate; Ca(OH)2: calcium hydroxide; TAP: triple antibiotic paste; and HE: hematoxylin and eosin stain.

Author (year)	Country	Sample size-tooth type	Age ranges (years)	Propolis format	Study against comparison groups (n)	Follow-up	Clinical and radiographic assessment: outcome assessed	Histological analysis (HE staining): outcome assessed	Key findings
Ahmad et al. (2022) [37]	Egypt	Forty sound premolars	15-25	Propolis powder mixed with ethanol	Propolis (20) against biodentine (20)	15 and 45 days	Post-operative pain.	Dentine bridge formation examination, structure of the odontoblast layer, and signs of inflammation.	After 15 days, the formation of dentine bridge was observed at the exposure site in the biodentine group while, in the propolis group, it was appeared after 45 days. Both groups exhibited mild inflammation.
Nasri et al. (2022) [38]	Iran	Forty one sound premolars	15-25	Propolis powder mixed with ethanol	Propolis (12) against MTA (12), biodentine (12), and negative control (5)	Eight weeks	Evaluation of pain, TAP, sinus tract presence, swelling, tooth mobility, and responses to thermal and tests for electrical pulp. Regarding the radiographic assessment, observation for periapical pathology.	Inflammation and dentine bridge formation evaluations.	MTA had (100%) success rate in clinical and radiographic outcomes in comparison to propolis and biodentine that had (91.7%). Biodentine showed the most inflammation, while MTA showed the least. Dentine bridge formation was lowest in the propolis group.
Mohanty and Ramesh (2020) [39]	India	One hundred two sound premolars	13-30	Propolis powder mixed with ethanol	Propolis (34) against MTA (34) and biodentine (34)	12 weeks	NR	Examination of dentin bridge formation.	78.8%, 93.5%, and 19.4% of participants showed dentine bridge formation in MTA, biodentine, and propolis groups, respectively. Dentin bridge formation was thinner in the propolis group.
Parolia et al. (2010) [40]	India	Thirty-six sound premolars	15-25	Propolis powder mixed with ethanol	Propolis (12) against MTA (12) and Ca(OH)2 (12)	15 and 45 days	Post-operative pain and sensitivity.	Dentine bridge formation assessment and inflammation signs.	MTA group showed improved control of inflammation and dentine bridge formation.

**Table 3:** Details of clinically selected studies in which propolis is used as an intracanal medicament in permanent teeth. NR: not reported; Y: years; MTA: mineral trioxide aggregate; Ca(OH)2: calcium hydroxide; TAP: triple antibiotic paste; VAS: visual analog scale; S1: first group sample; S2: second group sample; and S3: third group sample.

Author (year)	Country	Sample size-tooth type	Age ranges (years)	Propolis format	Study against comparison groups (n)	Follow-up	Assessment method: outcome assessed	Key findings
Lillygrace et al. (2021) [41]	India	Thirty non-vital young permanent teeth (single rooted with open apex)	7–14	Propolis paste: mixing propolis powder with saline.	Propolis (15) against TAP (15)	Three to four weeks	Bacterial colony counts at three stages: S1 (after access opening), S2 (after irrigation), and S3 (after three to four weeks of medicament placement).	TAP and propolis showed reduced bacterial counts (p < 0.05). TAP: counts dropped from 1906.75 (S1) to 817.25 (S3). Propolis: counts dropped from 1427.87 (S1) to 252.37 (S3). Statistically non-significant difference between propolis and TAP.
Shabbir et al. (2021) [42]	Pakistan	Eighty single-rooted necrotic teeth	20–40	NR	Propolis (40) against Ca(OH)2(40)	Four, 12, 24, 48, 72 hours, and four days post-operatively	VAS: It is used for pain intensity and is categorized into four levels: no/mild, moderate, severe, and extreme pain.	No significant pain score difference between age groups and tooth types. Half of the samples had higher pain scores on days two to four (p < 0.05). Most had no/mild pain.
Shabbir et al. (2020) [43]	Pakistan	Eighty single-rooted necrotic teeth	20–40	NR	Chinese propolis paste (40) against Ca(OH)2 (40)	Four, 12, 24, 48, and 72 hours post-operatively	VAS: It is used for pain intensity; flare-up incidence is defined as ≥20-point VAS increase between intervals.	No significant difference (p > 0.025) in pain scores between groups. In addition, >78% of patients in both groups had no/mild pain. Flare-ups: 14.8% overall; higher in propolis (17%) vs. calcium hydroxide (12%).
Tirukkolluru and Thakur (2019) [44]	India	Forty-five single-rooted teeth	35–50	Propolis powder mixed with moxifloxacin in saline.	Propolis with moxifloxacin (15) against TAP (15) and Ca(OH)2 (15)	Seven days	Bacterial colony-forming unit (CFU) counts before (S1) and after (S2) medicament placement.	All groups showed a significant reduction in microbial load (p < 0.05). No statistically difference in microbial reduction among all groups (p > 0.05).

*Primary* *Outcomes*

Table 2 provides a summary of four clinical trials that evaluated the use of propolis for dental capping in permanent teeth and comparisons with other materials such as biodentine, MTA, and Ca(OH)2. In these four RCTs, 219 sound permanent premolars were treated [37-40]. Propolis was primarily formulated as a powder mixed with ethanol [37-40] and used on healthy premolars in patients aged 13 to 30 years [39]. Follow-up ranged from 15 days [37] to 12 weeks [39]. Various assessment methods were used, including clinical examination, radiographic examination, histologic examination, VAS for pain intensity, and microbiologic analysis. Outcomes reported included clinical outcomes (pain after surgery, dental checkup and movement, illness, facial features) and histologic parameters (bridge construction and inflammation were observed in materials like biodentin after 15 days and propolis after 45 days and MTA).

Although propolis was found to be somewhat effective, one study reported that it has less tooth penetration and more associated inflammation than MTA or biodentine [39]. In particular, MTA has consistently had high success rates across RCTs [38-40]. Propolis had consistently thinner dentinal bridges, as reported by one study [39], and two studies reported poorer results in terms of inflammation, a reduced success rate of 91.6% compared to MTA (100%) [37,40] (Table 2).

*Secondary* *Outcomes*

**Figure 2:**
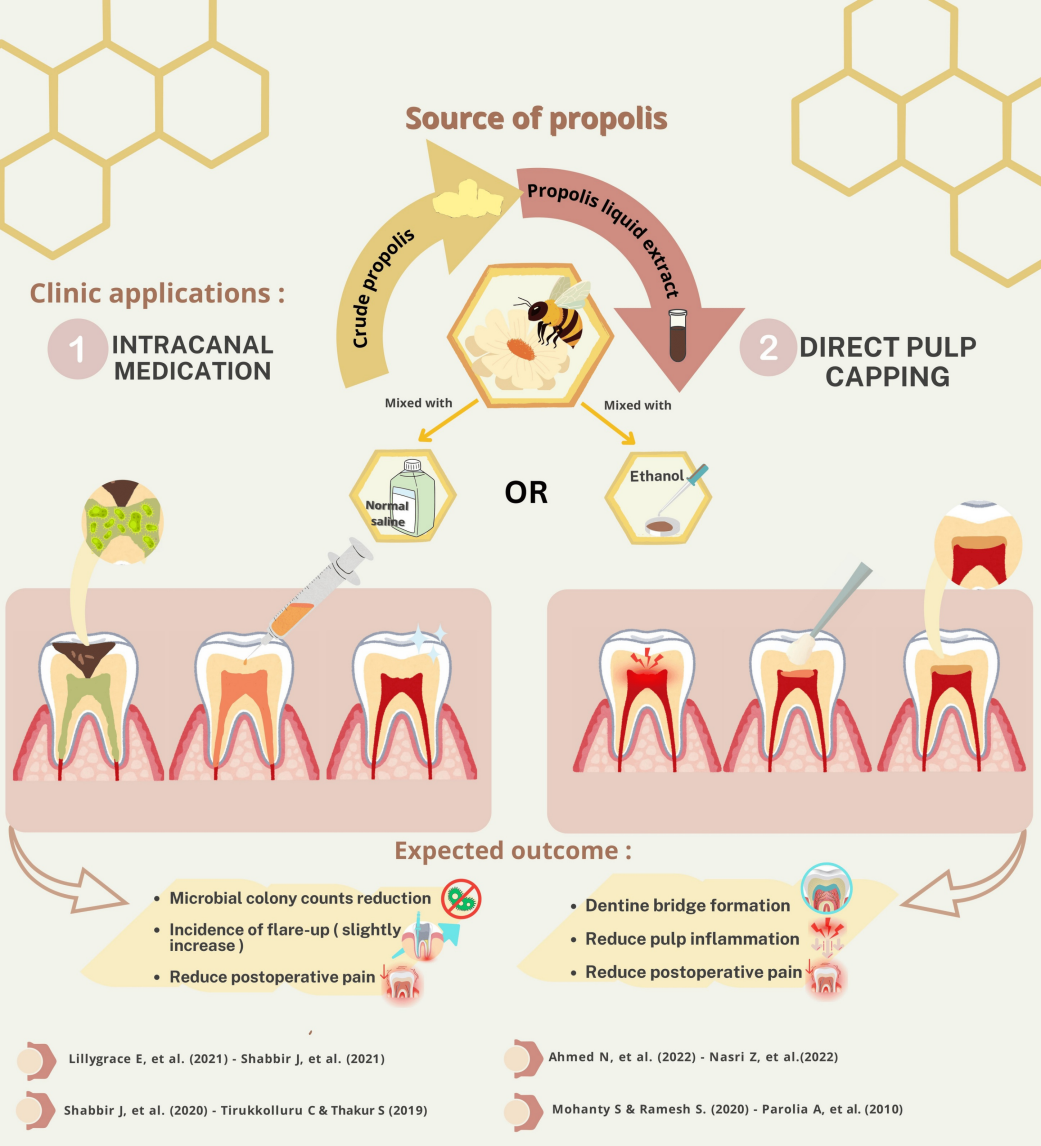
A summary of the different clinical applications of propolis used for endodontic treatment in the included studies. Reference sources: [37-44]. Note: This image is the author's own creation.

Table 3 summarizes four clinical trials investigating the use of propolis as an intracanal medicament in the treatment of permanent teeth. In these four RCTs, 235 sound single-rooted permanent teeth were treated [41-44]. Patient age ranged from seven [41] to 50 years [44]. Propolis was formulated as a powder mixed with saline [41] and antibiotics [44]. Propolis was compared with TAP [41,44] and Ca(OH)2 [42-44]. Microbiologic [41,44] and VAS assessments were utilized [23,43]. These studies suggested that propolis had an antimicrobial effect comparable to other materials like TAP. However, the difference was not statistically significant (p > 0.05). A summary of the different clinical applications of propolis used for endodontic treatment in the included studies is illustrated in Figure 2.

Grading of Recommendations Assessment, Development, and Evaluation (GRADE) Assessment

A GRADE was performed to evaluate the RCTs strengths and limitations in important areas. Some concern was observed in methodological limitations (RoB) as three of the eight included RCTs had a low RoB score. Similar findings were observed in the inconsistency domain. No concerns were identified in the remaining domains. The overall level of evidence was considered moderate RoB (Table 4).

**Table 4:** GRADE certainty of evidence. GRADE: Grading of Recommendations, Assessment, Development, and Evaluation; RCTs: randomized clinical trials.

GRADE domain	Judgment	Concerns	Level of evidence
Limitations in methodology (risk assessment)	Among eight RCTs, only three RCTs had low RoB [39,42,43], and three studies had unclear RoB [38,41,44], while two studies had high RoB [37,40] particularly, a study performed by [40] which had high RoB in three domains (random sequence, allocation, and incomplete outcome data).	Some concern	ƟƟƟ (Moderate)
Indirectness	Patients, interventions, and comparators in studies provide direct evidence of existing medical questions.	No concern	
Imprecision	Included studies performed proper statistical analysis.	No concern	
Inconsistency	Studies showed inconsistencies as RCTs did not report the propolis origin extraction method.	Some concern	
Publication bias	Even meta-analysis was not performed. However, both positive and negative outcomes (either effective or not effective compared to other materials) regarding propolis were published.	No concern	

The results of the GRADE assessment revealed a moderate level of evidence due to concerns related to risk of bias (RoB) and inconsistency across studies. Specifically, three of the eight studies had a low RoB, while three studies had an unclear RoB, and two had a high RoB, particularly in the random sequence generation, allocation concealment, and incomplete outcome data domains. These limitations, coupled with inconsistencies in reporting on propolis origin and extraction methods, resulted in a moderate rating for the overall body of evidence.

*Methodological* *Quality* *Assessment*

**Figure 3:**
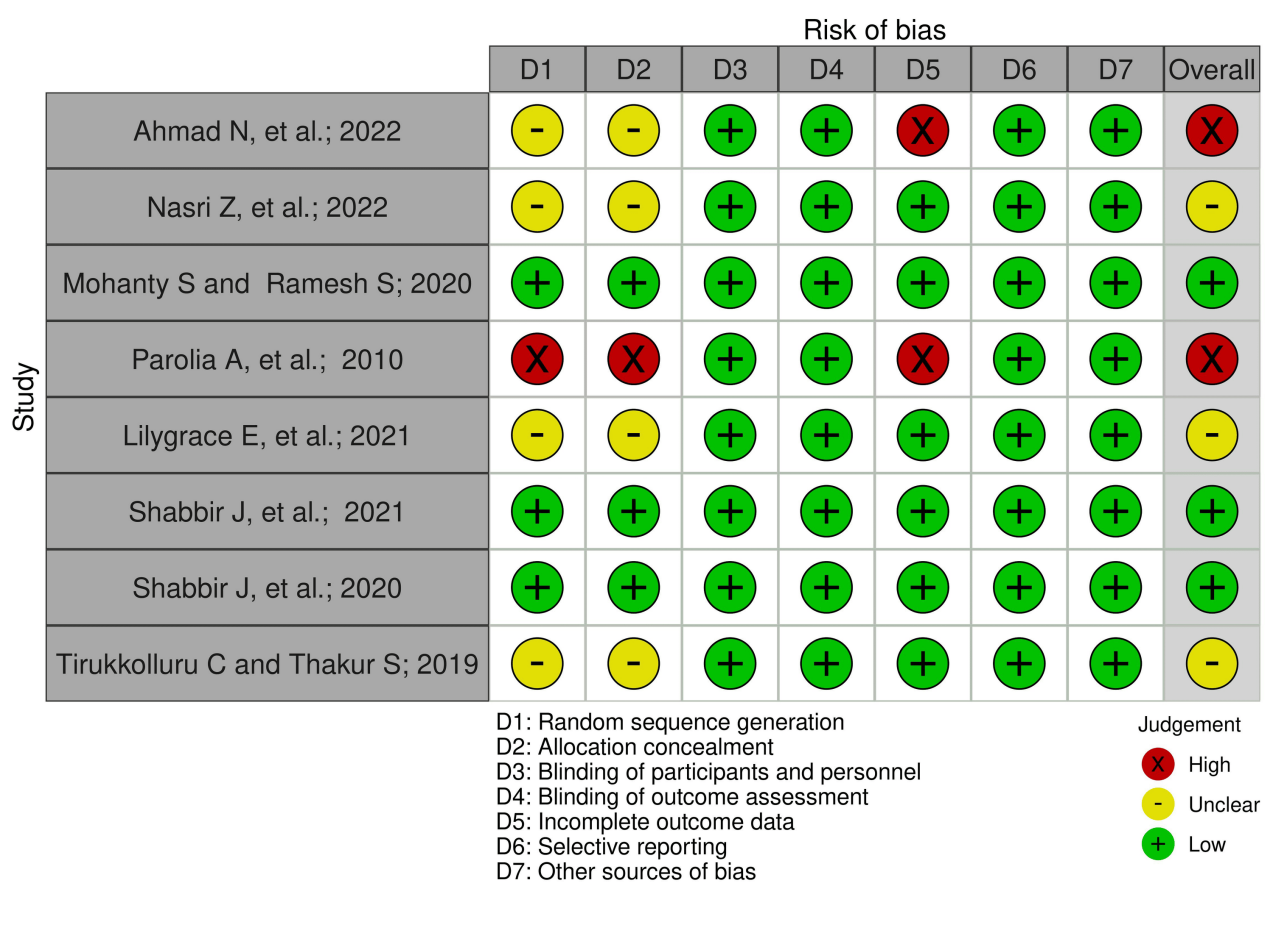
The individual methodological quality assessment of each included study. Reference sources: [37-44].

**Figure 4:**
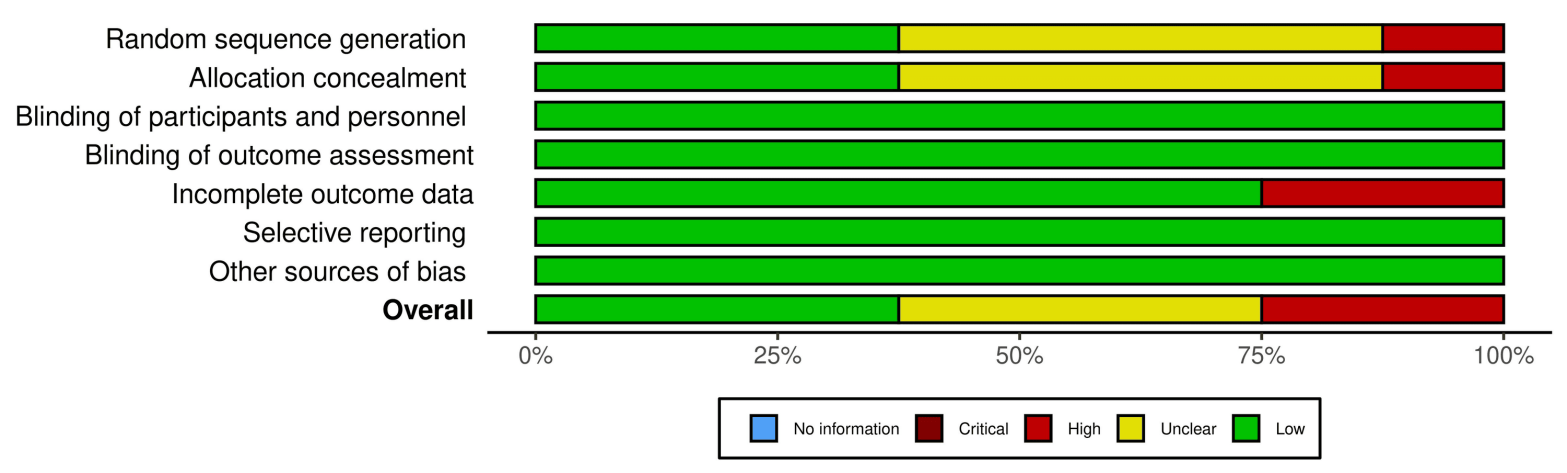
The overall methodological quality assessment of the included studies. Reference sources: [37-44].

Among the eight publications evaluated here, three were found to have a low RoB [39,42,43], three had an unclear RoB in the random sequence generation and allocation concealment domains [38,41,44], and two had a high RoB [37,40] due to random sequence generation, allocation concealment, and incomplete outcome data (Figures 3, 4).

The results of the risk of bias (RoB) and GRADE assessment were considered critical in evaluating the quality of the evidence in our review. According to the Cochrane RoB 2.0 tool, three studies showed a low risk of bias [39,42,43], while three had unclear risk in key areas like random sequence generation and allocation concealment [38,41,44]. Two studies had a high RoB, particularly in the random sequence generation, allocation concealment, and incomplete outcome data domains [37,40]. These factors contributed to concerns in the methodological limitations domain. The GRADE assessment also highlighted concerns about inconsistency, as some studies did not provide clear details about propolis origin and extraction methods. Overall, the evidence was rated as moderate due to these concerns, which may limit the generalizability of the findings.

Regarding the sources of funding for the included studies, this information was reviewed for potential bias. While the majority of the studies did not explicitly declare any conflicts of interest or funding sources, this may introduce a risk of bias in the interpretation of results. In this systematic review, the funding sources were not reported, and there were no potential conflicts of interest that could influence the findings of the included studies [37-44]. However, no direct evidence of funding-related bias was identified in the evaluation of study quality.

Discussion

Propolis has been reported in the dental literature as an effective material with antimicrobial, anti-inflammatory, therapeutic, and wound-healing properties [45,46]. This systematic review included all the available RCTs evaluating the role and application of propolis in the endodontic treatment of mature or immature permanent teeth of healthy patients.

*Use* *of* *Propolis* *as* *Pulp* *Capping* *Material* *in* *Permanent* *Teeth*

RCTs reviewed here show that permanent teeth treated with propolis used as a pulp capping material with other materials had less microleakage or the penetration of bacteria and fluids into the pulp chamber of the tooth and more inflammation than similar teeth treated with MTA or biodentine. In particular, MTA consistently had the highest success rates across RCTs. This difference may be due to their different physical, chemical, and biological properties.

MTA and biodentine are highly stable materials that are biocompatible, have sealing ability, promote dentin bridge formation, and are helpful in reducing inflammation [47,48]. Their ability to release calcium ions promotes mineralization and creates an alkaline environment, which aids healing and has antibacterial effects [49]. The consistent formulation and high bioavailability of MTA and biodentine contribute to their high success rates.

While propolis has antibacterial and antifungal properties, its performance may vary due to differences in composition, application, and inherent coatings [50]. The clinical application of propolis can be challenging due to an inconsistent formulation due to varying geographical origins [51] and extraction methods [52,53]. These variations present challenges in replicating its therapeutic effects and ensuring consistent product quality.

Our findings align with the findings of another study, which treated 42 molar teeth with MTA, light-cured calcium silicate, and propolis [54]. Teeth were evaluated clinically and radiologically three and eight months after treatment. The light-cured calcium silicate-treated group had the highest amount of dentin deposition, followed by MTA and propolis [54]. Similarly, 75 healthy children with dental caries in their primary molar teeth were treated with propolis, MTA, and biodentine. MTA and biodentine had a 100% success rate at three and six months of follow-up, while propolis had an 84% success rate [55]. In contrast, another study revealed that propolis had comparable cell viability when compared to MTA and demonstrated significantly higher mineralization and anti-inflammatory effects on human dental pulp cells (hDPCs) [56]. A review also reported that propolis was associated with promising clinical outcomes, including inflammation control, tissue enhancement, and root canal system disinfection [31]. These benefits have been attributed to the presence of natural substances found in the bee wax extract [57].

*Use* *of* *Propolis* *as* *Intracanal* *Medicament* *in* *Permanent* *Teeth*

RCTs reviewed here show that propolis used as an intracanal medicament in permanent teeth was associated with improved efficacy and VAS scores. That impact was not significantly different from other materials like TAP and Ca(OH)2, possibly due to the antimicrobial properties of TAP, Ca(OH)2, and propolis. Our findings are in agreement with an RCT of 46 patients treated with propolis or Ca(OH)2 as intracanal medicaments, and no difference in post-operative VAS scores was observed during follow-up [58].

Fifty dentine discs were used for the antimicrobial assessment of propolis-based and Ca(OH)2 intracanal treatments. It was found to be significantly more effective than non-setting Ca(OH)2 after short-term application [59]. Furthermore, when propolis was compared with Ca(OH)2, chlorhexidine gel, and a combination of propolis plus Ca(OH)2, all were found effective against *E. faecalis* [60].

The evidence reviewed here is insufficient to recommend the widespread use of propolis in endodontic treatment at this time. However, the promising non-significant reports support further research into the chemical composition, concentration, and formulation of propolis extracts in order to better evaluate their potential in endodontic treatments. Development of a standardized propolis formulation in a comprehensive clinical evaluation, especially in primary teeth, is necessary before considering its application in permanent teeth. Further clinical trials are indicated.

*Study* *Strengths* *and* *Limitations*

A strength of this systematic review is its inclusion of recent RCTs on this topic, as no previous systematic reviews have been published in endodontics. Several limitations were identified in this review. Only eight RCTs were identified, and although these studies provide useful insights, their findings may not be directly applicable to clinical practice. This review is based solely on the four largest scientific databases, potentially excluding relevant studies not indexed in these databases. Furthermore, the propolis-related variables, including their origin, composition, and extraction method, were inadequately mentioned in the selected articles. This lack of reporting limits the reliability of the results. In addition, there was significant variability in how propolis was formulated in the studies, with extracts typically mixed with ethanol, saline, or moxifloxacin in saline, and with different concentrations and ratios. This clinical variability may have impacted the effectiveness of propolis.

## Conclusions

This systematic review suggests that propolis improves dentin formation, reduces microbial infections, and alleviates post-operative pain. However, the studies showed that the difference was non-significant when compared to other materials like biodentine, Ca(OH)2, and TAP. Therefore, its application is not recommended as a definitive treatment due to the limited evidence and variability in the clinical outcomes across studies, even though propolis has been confirmed as a safe material for pulp capping and as an intracanal medicament in endodontic treatments for both vital and non-vital pulp conditions in permanent teeth. Further research is needed to standardize its formulation and evaluate its effectiveness.
